# Continuous erector spinae plane block for analgesia of sternum closure using a latissimus dorsi muscle flap for mediastinitis after coronary artery bypass grafting: a case report

**DOI:** 10.1186/s40981-020-00370-3

**Published:** 2020-08-19

**Authors:** Yu Yamane, Masayuki Kosaka, Haruki Akiizumi, Mitsuo Kuroda

**Affiliations:** Department of Anesthesiology, Hyogo Brain and Heart Center, 520 Saisho-Koh, Himeji, Hyogo Japan

**Keywords:** Continuous erector spinae plane block, Latissimus dorsi muscle flap, Coronary artery bypass grafting, Antithrombotic drug

## Abstract

**Background:**

Erector spinae plane block (ESPB) is useful for providing analgesia after thoracic surgery. Previous reports show that ESPB is safely performed in patients receiving antithrombotic drugs. We effectively performed continuous ESPB in a patient receiving aspirin after coronary artery bypass grafting.

**Case presentation:**

A 62-year-old man with mediastinitis was scheduled for sternum closure using a latissimus dorsi muscle flap. He had gone coronary artery bypass grafting and was taking aspirin. After induction of general anesthesia and tracheal intubation, a catheter was inserted for ESPB from the T6 level under ultrasound monitoring and infusion of ropivacaine was started. Tracheal tube was removed in the operating room, cold sense was absent between T2–8, and analgesia was between T3–T8 after uneventful surgery. There were no complications associated with ESPB postoperatively.

**Conclusion:**

Continuous ESPB was a safe and useful analgesic method in a case undergoing sternum closure using a latissimus dorsi muscle flap.

## Background

After initial report by Forero et al. in 2016 [1], erector spinae plane block (ESPB) has been widely used for analgesia after thoracic surgery [2–4]. For ESPB, local anesthetics are administered to the erector spinae muscles away from the spinal cord and nerve roots compared with epidural anesthesia and are unlikely to cause complications [1, 5]. Previous reports show that ESPB is safely performed in patients receiving antithrombotic drugs [6, 7]. We report a case in which ESPB was safely performed and useful for postoperative analgesic management of sternum closure using the latissimus dorsi muscle flap for mediastinitis after coronary artery bypass grafting (CABG).

## Case presentation

A 62-year-old man (175.6 cm 75 kg) was scheduled for sternum closure using the right latissimus dorsi muscle flap for mediastinitis. He had gone CABG using the bilateral internal thoracic arteries 8 months before. He underwent debridement and vacuum-assisted closure (VAC) of the infected wounds 1 month after surgery. Although sternum closure using an omental flap was performed thereafter, debridement and VAC were reintroduced because of infection. His medical history included diabetes mellitus with end-stage diabetic nephropathy. He has been receiving hemodialysis, and the right lower leg amputated for diabetic gangrene. He was receiving linagliptin and mitiglinide calcium hydrate and aspirin. His laboratory data showed renal dysfunction (estimated glomerular filtration rate 6.2 ml/min) and anemia (hemoglobin 9.6 d/dl), but no abnormal coagulation was observed, and the platelet count was 145,000/μl. He also complained of postoperative nausea and vomiting (PONV) probably due to fentanyl after multiple anesthesia histories.

Considering the possibility of widespread surgical wound and he was taking aspirin, continuous ultrasound-guided ESPB was planned. Aspirin was continued until the day of surgery. He received no premedication. Standard monitoring and intra-arterial blood pressure monitoring were performed. General anesthesia was induced with propofol 70 mg, fentanyl 250 μg, and rocuronium 60 mg and maintained with 1.5% sevoflurane in an inhaled oxygen concentration of 60% with a total flow of 3 l/min and remifentanil 0.1–0.2 μg/kg/min. After tracheal intubation, he was placed in the left lateral decubitus position. After performing standard skin asepsis, the linear transducer (12 MHz, LOGIQ e; GE Healthcare, Chicago IL), within a sterile sleeve, was positioned on the patient in a transverse orientation and the T6 spinous process image was taken. The transducer was moved right laterally to identify the transverse process of T6 and then rotated 90° to visualize the right transverse process and the erector spinae muscles above it. A continuous nerve block set (Contiplex Tuohy Ultra set; B BRAUN, Melsungen, Germany) with an 18-G, 100-mm Tuohy needle was inserted in-plane in a caudal to cranial direction until contact with the right T6 transverse process was made. After confirming that there was no reverse blood flow, hydrodissection with 2 ml of 0.375% ropivacaine was used to confirm the correct needle tip position. Eighteen milliliters of 0.375% ropivacaine was injected, and a 20-G catheter was advanced through the needle. After injecting 20 ml of 0.375% ropivacaine again from the catheter 55 min after the initial injection, 0.2% ropivacaine was continuously infused at 5 ml/h before the start of surgery. Hemodynamic stability was achieved without the need for an additional bolus administration of fentanyl during surgery. One gram of acetaminophen was intravenously infused 30 min before the end of the operation. There were no other events of note during the operation. The operative time was 5 h and 49 min, and the anesthesia time was 8 h and 2 min.

After extubation in the operating room and admission to the intensive care unit (ICU), the nerve blockade area and Numerical Rating Scale (NRS) were confirmed when sufficient consciousness was reached. Regarding nerve blockade, the cold sign was T2–T8, pinprick was T3–T8, and NRS at rest was 1/10 immediately after surgery. The main pain area was the wound where the right latissimus dorsi muscle flap was collected, but pain of the median sternum wound was not noted. Acetaminophen at 500 mg was administered orally because the NRS temporarily increased to 7/10 due to body movement. However, the NRS at rest was 1–2/10 and that while active was 4/10. For pain during movement, 3 ml of patient-controlled analgesia (PCA) consisting of 0.2% ropivacaine was flushed, and this was performed twice in the ICU. After leaving the ICU 24 h after the operation and being transported to the general ward, pain was not aggravated and the ESPB catheter was removed 56 h after the operation. After removal of the catheter, oral administration of acetaminophen during pain provided good pain control. No ESPB-related complications were observed during ESPB catheter placement or after removal. Regarding PONV, nausea was observed immediately after admission to the ICU and only a single intravenous injection of 10 mg of metoclopramide was administered. Thereafter, the patient had no PONV.

## Discussion

We report a case in which continuous ESPB was safe and useful for the postoperative analgesic management of sternum closure using a latissimus dorsi muscle flap in a patient with mediastinitis after CABG taking aspirin. Thoracic epidural anesthesia (TEA) and thoracic paravertebral block (TPVB) have extensively been used for analgesia after thoracic surgery. In the present case, epidural anesthesia was avoided because the patient was receiving aspirin. Absence of cold sense by TPVB is limited to a median of only four dermatomes after continuous infusion of ropivacaine at 6 ml/h for 24 h, regardless of its concentration [8], which would have been insufficient for providing adequate anesthesia in our case. The latissimus dorsi muscle collection site was a wide wound, and in this case, dermatome spanned from T3 to T9. In addition to the above reasons, there was also a report that good ESPB analgesia in single dose was obtained for breast reconstruction using the latissimus dorsi muscle flap [3], and continuous ESPB was indicated in this case.

ESPB is a relatively new peripheral nerve block reported by Forero et al. [1] in 2016 as an analgesic method for chronic pain. Several reports [2–4] demonstrated it to be useful as a postoperative analgesia method for thoracic surgery. In addition, ESPB may be useful for patients with abnormal coagulation. The American Society of Regional Anesthesia and Pain Medicine guidelines [9] suggest management based on site compressibility, vascularity, and consequences of bleeding should it occur. To that point, Chin et al. [5] describe the ESPB as being remote to major nervous and vascular structures thereby minimizing risks of clinically significant hematomas in patients with abnormalities of coagulation. The erector spinae muscles can be compressed in the unlikely event of hematoma formation [7]. In fact, it has been reported that patients who have been taking antiplatelet drugs were safely performed ESPB [6, 7]. For this reason, compared with TEA and TPVB, the risk of neuropathy due to bleeding complications may be low.

In a report summarizing the range of effects of ESPB [10], the amount of local anesthetic required to block one dermatome was reported to range widely from 2.5–6.6 ml, with a median value of 3.4 ml. ESPB is expected to achieve a wide area of nerve blockade in proportion to the volume compared with TPVB. In this case, an increased local anesthetic volume resulted in extensive nerve blockade and a good NRS. Fentanyl was used only at a dose of 250 μg at the time of introduction, and no opioid was used as postoperative analgesia. Therefore, nausea was observed immediately after admission to the ICU, and thereafter, no PONV was observed. The analgesic management by ESPB was considered to play a role in the reduction of PONV.

## Conclusion

We reported a case in which ESPB was safe and useful for postoperative analgesia management of sternum closure using the latissimus dorsi muscle flap in a patient with mediastinitis who was taking aspirin after CABG.

## Data Availability

Not applicable

## Abbreviations

ESPB: Erector spinae plane block
CABG: Coronary artery bypass grafting
VAC: Vacuum-assisted closure
PONV: Postoperative nausea and vomiting
ICU: Intensive care unit
NRS: Numerical Rating Scale
PCA: Patient-controlled analgesia
TEA: Thoracic epidural anesthesia
TPVB: Thoracic paravertebral block
